# Role of Mitophagy in the Pathogenesis of Stroke: From Mechanism to Therapy

**DOI:** 10.1155/2022/6232902

**Published:** 2022-02-27

**Authors:** Wei-Jie Zhong, Xiao-Sheng Yang, Han Zhou, Bing-Ran Xie, Wen-Wu Liu, Yi Li

**Affiliations:** ^1^Department of Neurosurgery, Ninth People Hospital Affiliated to Shanghai Jiao Tong University School of Medicine, Shanghai 200011, China; ^2^Naval Characteristic Medical Center Diving and Hyperbaric Medicine Research Laboratory, Shanghai 200433, China

## Abstract

Mitochondria can supply adenosine triphosphate (ATP) to the tissue, which can regulate metabolism during the pathologic process and is also involved in the pathophysiology of neuronal injury after stroke. Recent studies have suggested that selective autophagy could play important roles in the pathophysiological process of stroke, especially mitophagy. It is usually mediated by the PINK1/Parkin-independent pathway or PINK1/Parkin-dependent pathway. Moreover, mitophagy may be a potential target in the therapy of stroke because the control of mitophagy is neuroprotective in stroke *in vitro* and *in vivo*. In this review, we briefly summarize recent researches in mitophagy, introduce the role of mitophagy in the pathogenesis of stroke, then highlight the strategies targeting mitophagy in the treatment of stroke, and finally propose several issues in the treatment of stroke by targeting mitophagy.

## 1. Introduction

Stroke is an acute cerebrovascular event that is one of the leading causes of death worldwide and a leading cause of adult disability [1]. In addition, it has a high recurrence rate, increasing the economic burden on the family and society (S. [2]). Due to the development of therapeutic strategies, early diagnosis and therapy [3], the reinforcement of patients' education on stroke prevention [4], and the introduction of rehabilitation center [5] and stroke unit, the mortality, and disability of stroke tend to reduce in recent years.

According to the cause of poor blood flow in the brain, stroke can be divided into two main types: hemorrhagic stroke and ischemic stroke [6]. Cerebral ischemia is a common type of stroke and accounts for 85% of strokes. Cerebral ischemia can be further subdivided into global and focal zones based on the extent of cerebral ischemia [7]. Although numerous studies have been conducted to investigate the therapies for stroke, no effective strategies have been developed so far. Currently, reperfusion therapy with thrombolytic agents such as mechanical thrombolysis (MT) or intravenous tissue plasminogen activator (tPA) is the only approved treatment for ischemic stroke. [8]. Surgical decompression is a widely life-saving therapy for hemorrhage stroke [9]. However, there are still limitations in the existing treatment [10]. Thrombolytic therapy is limited in clinical practice due to its short time window [11] and has the risk of causing intracranial hemorrhage (ICH), which may worsen the neurological impairment and even cause death. Therefore, more studies are needed to investigate the pathogenesis of stroke, which may help the development of new therapies for stroke.

Mitochondria are known as a “power factory” and crucial organelles for aerobic respiration, which may provide energy for intracellular activities. In the normal cells, mitochondria continuously function to metabolize oxygen. Mitochondria are a major source of intracellular reactive oxygen species (ROS) generation as a result of the oxygen metabolism [12]. Oxidative stress occurs due to an imbalance between the generation and detoxification of ROS. At physiological level, ROS may serve as redox signaling molecules, which can transduce signals from the mitochondrial compartment to other compartments of the cell. In case of oxidative stress, excessive formation of ROS in the mitochondria may lead to the dysfunction of oxidative phosphorylation and mitochondrial morphology, leading to several complications. Once this cycle starts, it does not stop (known as the vicious cycle of the mitochondria) and finally causes mitochondrial dysfunction. The mitochondrial dysfunction will ultimately cause the depletion of ATP, influx in calcium, and the opening of the mitochondrial permeability pore, eventually leading to apoptosis or even cell death [13]. The energy depletion of brain cells is the first link of ischemic stroke [14]. The neurons are susceptible to the ROS-induced damage because they require high energy and have large numbers of mitochondria, weak antioxidant defense, and weak bioavailability to antioxidant treatment and molecules. Oxidative stress due to the excessive ROS production plays an essential role in the fundamental pathologic progression of brain injury after stroke [15]. A variety of studies have confirmed that mitochondrial dysfunction is closely related to cerebral ischemia-reperfusion (I/R) injury [16] because mitochondrial dysfunction is a key point leading to cell damage during I/R injury in the brain and is linked to the death of nerve cell during stroke [17].

Mitophagy is a type of selective autophagy that can maintain normal physiological processes by selectively removing damaged or dysfunctional mitochondria. In 1966, mitophagy was first identified by electron microscopy (EM) [18]. In 2005, Lemasters proposed the term “mitophagy” which plays a pivotal role in the accumulation of mtDNA somatic mutations, being delayed with aging [19]. Since the proposal of the term mitophagy, increasing studies have been conducted to investigate the role of mitophagy during neurological diseases, such as stroke, Parkinson's disease (PD), and traumatic brain injury (TBI) [20].

Herein, we give a brief summary of recent advances in mitophagy to introduce the association between mitophagy and stroke and then propose strategies targeting mitophagy in the treatment of stroke.

## 2. Autophagy

### 2.1. Introduction of Autophagy

Over the past years, the concept of autophagy as a nonselective system has been repudiated, and recently, more important researches open the field of selective autophagy ([21]). Nonselective autophagy involves processing cytoplasmic components in a relatively nonselective manner, such as microautophagy and macroautophagy [22]; its degradation in the lysosome produces nutrients necessary during starvation. Instead, selective autophagy recognizes autophagy substrates through specialized receptors [23].

Autophagy is a ubiquitous process in which cellular materials are transported to the lysosome for degradation, which allows the basic turnover of cellular components and affords energy and macromolecular precursors [24]. Autophagy comes from the Greek and means “to eat oneself” and was first proposed in 1967, originating in the observed degradation of intracellular structures in lysosomes [25]. Autophagy is a dynamic, multistage cellular physiological process, the regulation of which has the involvement of multiple genes, and the activity of autophagy is largely assessed by multiple detection methods [26]. So far, the detection of LC3B-II has been processed through fluorescence staining or Western blotting (WB) and direct observation of autophagosomes by electron microscopy (EM) has become the main methods for autophagy activity assessment.

### 2.2. Autophagy Recognition

50 years ago, autophagy was recognized by transmission electron microscopy (TEM) for the first time [27]. It is also a sensitive technique to detect autophagic vesicles, as early autophagosomes contain intact cytoplasm or organelles, and late autolysosomes contain partially degraded cytoplasm and organelle materials [28]. The assessment of autophagic activity by TEM is not objectively quantitative [29]. Researchers have used the characteristic molecules formed by autophagosomes to derive more quantitative assays for autophagic assessment [30]. Studies have shown the importance of light chain 3 (LC3; a homolog of yeast Atg8) in the formation and function of autophagosomes, and so, LC3-I and LC3-II antibodies are widely used to identify autophagy [31, 32]. Other molecules are involved in targeting cargo to autophagosomes or activating autophagy, such as Atg7, Atg3, and Atg16L. [33]. In addition to EM and WB, past studies of autophagy have involved fluorescence microscopy [34]. Particularly, green fluorescent protein (GFP) is fused to the amino-terminal surface of LC3 and is widely used to detect autophagy [35]. The rate of autophagy flux has also been used as a meaningful method for assessing autophagy activity. The long-lived protein was also employed to assess the autophagic flux [36], such as betaine homocysteine methyltransferase (BHMT) [37], Beclin-1 [38], and polyQ80-luciferase [39].

## 3. Mitophagy

### 3.1. Introduction of Mitophagy

Mitochondria are dynamic organelles that undergo continuous events of biogenesis, remodeling, and turnover. The selective autophagic degradation of dysfunctional mitochondria, termed mitophagy, can ultimately eliminate dysfunctional mitochondria to maintain normal physiological processes, which is one of the quality control mechanisms in cells and can protect cells from injury. It has been reported that mitophagy can be induced by multiple triggers, including hypoxia, stroke, normal cell process, viral infection (L. [40]), and ROS [41]. According to the inducements, mitophagy could be divided into three types: basic, progressive, and stimulus-induced [42]. Basal mitophagy refers to the process that cells degrade abnormal or aging mitochondria under physiological conditions. Progressive mitophagy is known as mitophagy occurring in different cell types during development, such as reticulocyte maturation [43]. Stimulus-induced mitophagy may cause the rapid degradation of mitochondria after severe extracellular stress, such as hypoxia, stroke, and redundant ROS. ROS is a major trigger of mitophagy. The eukaryotic cells have developed multiple mechanisms to abrogate or ameliorate ROS production through the evolutionary refinement. At pathological circumstances, excessive ROS cannot be effectively neutralized by the antioxidants and antioxidases and may cause the damage to mitochondria and other cell components. Therefore, cells can initiate the selective degradation of damaged or impaired mitochondria (mitophagy) as an additional mechanism to deal with ROS [44].

### 3.2. Mitophagy Recognition

The removal of impaired mitochondria is of great importance for the prevention of cellular death. Methods commonly used to investigate cellular mitophagy include EM, fluorescence microscopy, and WB for the detection of proteins related to mitochondrial degradation [45]. Some recent studies have proposed new methods for the detection of mitochondrial autophagy, such as MitoTimer, mt-Keima, and mito-QC [46]. Thus far, various results should still be comprehensively evaluated in the monitoring of mitophagy.

EM provides visualization of mitochondria that are surrounded or engulfed by autophagic membranes [47]. It helps understand the relationship between mitochondria and other structures. Late mitophagy can be distinguished by the presence of mitochondria in different degradation stages in a single membrane, and early mitophagy is identified by recognizing the mitochondrial spines and other structures inside the double-membrane autophagosomes [46]. However, due to the morphological changes and degradation of mitochondria in the process of mitophagy, the relatively late mitophagy is not easily recognized in EM [47]. In addition, this technique requires sophisticated equipment and experience and is extremely difficult to quantify accurately.

Fluorescence microscopy is a useful tool that can quantitatively assess mitophagy. According to fluorescence microscopy, GFP-LC3 is used to label autophagosomes, or the MitoTracker colabeling method is employed to mark the colocalization of mitochondria and autophagosomes ([48]). However, quantifying the colocalization of autophagosomes or lysosomes between mitochondria is subjective and still requires consistent standards throughout the experiments [46].

In addition, the detection of proteins related to mitochondrial degradation by immunohistochemistry or Western blotting can be employed to determine the activity of mitophagy [45]. However, the positive results largely rely on a high level of mitophagy because it is impossible to identify whether the protein is degraded by internal mitochondrial proteases or the entire mitochondria is degraded by lysosomal delivery and makes the data cumbersome to interpret ([48]).

Several recently developed techniques for the identification of mitophagy, such as MitoTimer, it is a timer fluorescent protein that targets the region encapsulated by the inner mitochondrial membrane [49]. MitoTimer is a new tool for the monitoring of mitochondrial aging renewal and can be used to access individual mitochondria or groups of mitochondria [50]. Similar to MitoTimer, Mt-Keima targets the mitochondrial matrix by fusing with COX8 [51] and is based on the pH-dependent fluorescence measurement of the coral-derived protein Keima. Similarly, Mito-QC is also pH-sensitive. The difference is Mito-QC fused with the mitochondrial-targeted sequence of mitochondrial outer membrane protein FIS1 [52]. Interestingly, for Mito-QC, the mCherry signal is more stable during mitophagic activation [45]. Although these tools are valuable for monitoring and quantifying mitochondrial autophagy, they also have their disadvantages. For example, Mito-QC targets the mitochondrial outer membrane, which could also be disassembled by the proteasome [45]. Longer exposure in the imaging process affects the results of Mt-Keima [46]. Besides, changes in MitoTimer fluorescence are not specific to mitochondrial degradation. MitoTimer needs to be combined with other methods to identify mitophagy [53].

### 3.3. Pathways Related to Mitophagy

**Figure 1:**
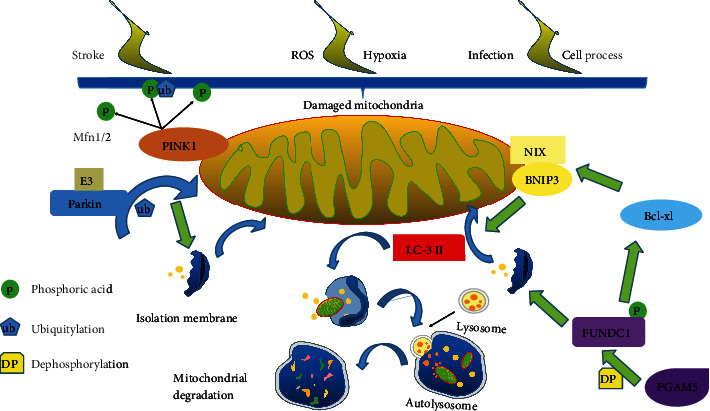
(a) When mitochondria are damaged, the PINK1 accumulates and recruits the E3 ubiquitin ligase Parkin from the cytosol especially to the damaged mitochondrion. Parkin ubiquitylates mitochondrial proteins and causes mitochondria to become engulfed by isolation membranes that then fuse with lysosomes. (b) BNIP3 and NIX are upregulated in response to hypoxia and reticulocyte maturation, respectively. These mitophagy receptors directly bind LC3 via LIR domains and induce isolation membrane recruitment for mitophagy. (c) FUNDC1 can bind LC3 to induce mitochondrial engulfment. (d) Finally, the mitochondria are sealed by the isolation membranes and fuse with the lysosome to be degraded.

To date, the identification of numerous receptors and adaptors underscores the existence of complex mechanisms that control the quality and quantity of mitochondria [42]. The pathways involved in mitophagy can be divided as PINK1/Parkin-dependent or independent ones (Figure 1).

#### 3.3.1. PINK1/Parkin-Dependent Mitophagy

Parkin and tensin homolog- (PTEN-) induced kinase 1 (PINK1) are known as key points in the parkinsonian syndrome [54]. After 2004, researches on mice conformed to the hypothesis of the PINK1/Parkin pathway related to mitochondrial quality control [55]. After 2015, many investigators found that the key molecule during mitophagy is the serine/threonine kinase PINK1 [56, 57].

PINK1/Parkin-dependent mitophagy is initiated with the activation of PINK1 [58]. After PINK1 is exposed to the cytosolic surface or senses mitochondrial damage signaling, PINK1 selectively activates and directs Parkin to the mitochondria on which PINK1 accumulates. For PINK1, two important phosphorylation processes are required for PINK1-induced Parkin activation. One is S65 in the Parkin Ubl domain, and the other is a similar S65 residue on ubiquitin [59, 60]. The phosphorylated PINK1 may induce Parkin's E3 ligase activity [61]. After that, Parkin ubiquitinates several mitochondrial proteins including mitofusins (Mfn), and causing mitochondria are swallowed by the separation membrane and then fuse with lysosomes [62].

Of note, some details are still not resolved fully. For example, how PINK1 precisely activates and directs Parkin selectively to the mitochondria is not clear completely, and how Parkin promotes mitophagy is also poorly understood.

#### 3.3.2. PINK1/Parkin-Independent Mitophagy

NIP3-like protein X (NIX; also known as BNIP3L), Bcl-2/E1B-19 KD-interacting protein 3 (BNIP3), and FUN14 domain containing 1 (FUNDC1) can interact directly with LC3 and GABARAP on the autophagosomal membrane without ubiquitination, mediating mitophagy [63]. Besides, they are outer mitochondrial membrane proteins and can be known as mitophagy receptors, which mediate mitochondrial clearance in response to stresses [64, 65].

NIX is a homolog of BNIP3, a proapoptotic mitochondrial protein [7]. NIX/BNIP3L and BNIP3 are involved in hypoxia-induced mitophagy [66]. Indeed, NIX and BNIP3 are transcriptionally regulated through forkhead box O3 (FOXO3) or hypoxia-inducible factor (HIF) [67, 68]. In addition, BNIP3 has been shown to enhance the binding of LC3-B and Gate-16 through phosphorylation at Ser17 and Ser24, inducing mitochondrial autophagy [69]. It has been revealed that red blood cells lose their mitochondria during differentiation by mitophagy [62]. NIX is required for mitochondrial removal and increases during the differentiation of red blood cells. NIX binds to LC3 on the isolation membrane and mediates the binding and sequestration of mitochondria into the autophagosome [70].

FUNDC1 has three transmembrane domains ([71]) and has been reported to play an important role in mitochondrial phagocytosis [72]. It contains a classical LC3-interacting region (LIR) and directly binds to LC3 or ATG8 to activate subsequent mitophagy [73]. Inhibitation of FUNDC1 or mutation of LIR motif can inhibit mitophagy when there is oxygen deficiency and blood loss [72].

### 3.4. Regulation of Mitophagy

Increasing studies have revealed that mitophagy is a dual character: mitophagy at the physiological level is essential for multiple cellular processes, but excessive activation of mitochondrial autophagy is also harmful to cellular hemostasis [74]. Impaired mitophagy is considered to be an important link leading to many pathological conditions. Therefore, activation or inhibition of mitophagy is essential for mitochondrial homeostasis.

#### 3.4.1. Activators of Mitophagy

Since mitophagy mainly functions to clear dysfunctional or damaged organelles, how mitophagy is initiated is a critical issue in studies on mitophagy. Therefore, it is necessary to study the pharmacological effects of promoting the clearance of dysfunctional or damaged organelles [75]. The activators of mitophagy can maintain mitochondrial homeostasis through different mechanisms. Currently, the activators of mitophagy can be divided into natural compounds and artificial chemical compounds. Resveratrol, as a natural compound, is a stilbene compound produced by several plants when they are injured or attacked by pathogens [76]. Resveratrol possesses a wide range of biological properties including antioxidative, anti-inflammation, neuroprotective, anticancer, and antiaging activities in various *in vitro* and *in vivo* studies [77]. It has been reported that resveratrol can activate the BNIP3-related mitophagy through HIF1 and 5′AMP-activated protein kinase (AMPK) ([78]). Li et al. found that rapamycin significantly enhanced mitophagy in a mouse model of spinal cord injury by increasing the translocation of damaged mitochondria by p62 and Parkin ([79]). As an artificial chemical, PMI can inhibit the protein-protein interaction (PPI) between the transcription factor Nrf2 and its negative regulator Keap1 [80]. PMI causes nuclear accumulation of Nrf2 and transcriptional activation of p62 recruitment. Then, the redox state of mitochondria changes, mitophagy is initiated, and mitochondria are targeted to autophagosomes through p62 [81]. Pearson et al. found that the Clec16A-Nrdp1-USP8 complex relies on ubiquitin signaling to promote mitochondrial autophagy and maintain mitochondrial quality control required for optimal *β* cell function [82]. In addition to the natural and artificial chemical compounds, several medicines can also induce mitophagy. Valinomycin is a respiratory chain inhibitor which has been reported to induce mitophagy by the PINK1/Parkin signaling pathway [83]. Metformin, a classical antidiabetic drug, may induce mitophagy by increasing Parkin activity and downregulating p53 expression [84].

#### 3.4.2. Inhibitors of Mitophagy

Under physiological conditions, the activation and inhibition of mitophagy remain stable, which may maximize the reuse of substances in cells without damaging cells. Thus, inhibition of mitophagy is also important for cellular physiology. The most commonly used regimens for *in vitro* inhibition of mitophagy rely on pharmacological inhibition of lysosomal acidification with the lysosomal reagents chloroquine and hydroxychloroquine or with the v-ATPase (vacuolar H+-ATPase) inhibitor bafilomycin A1 [85]. In addition, the activation of mitophagy mainly depends on ubiquitination, and USD15/35/30 may exert negatively regulated mitophagy via direct deubiquitination effects on the Parkin substrates [86–88]. Mdivi-1 is known as a small molecular inhibitor of dephosphorylating dynamin-related protein 1 (Drp-1). Studies have shown that inhibiting Drp1 can suppress mitochondrial breakage and activate apoptosis ([89]). Wu et al. found that Mdivi-1 treatment inhibited mitophagy by inhibiting the upregulation of PINK1 and Parkin expression [90].

## 4. Mitophagy and Stroke

It has been confirmed that the imbalance of activation and inhibition of mitochondria autophagy is involved during some diseases, including stroke. As mentioned above, mitophagy has also been found as a double-edged sword in the pathogenesis of stroke (Table 1), and inadequate removal of impaired mitochondria or excessive degradation of essential mitochondria can be harmful to cellular hemostasis [74].

### 4.1. Induction of Mitophagy

Some investigators have speculated that mitophagy induction is beneficial for stroke [104].

#### 4.1.1. In Vitro

In oxygen-sugar deprivation/reoxygenation (OGD/R) of hippocampal neurons, hydrogen (H_2_) could activate mitophagy. In addition, H_2_ has a neuroprotective effect on neurons, which is associated with enhanced PINK1/Parkin-mediated mitochondrial autophagy [105]. Apelin-36 has been reported as a neuropeptide with protective effects against brain I/R injury [106]. In HT22 cells of OGD/R, SIRT1-mediated mitophagy is involved in the neuroprotective effect of Apelin-36, which is closely related to the PINK1/Parkin pathway [107]. StigmasterolIn (ST) is involved in neuronal development [108]. There is evidence that ST shows a promising neuroprotective effect by attenuating GluN2B-mediated excitatory toxicity and oxidative stress and inducing mitochondrial autophagy in hippocampal neurons exposed to hypoxia/reoxygenation (H/R) [109]. In rat cortical neurons, Ye et al. found that resveratrol could alleviate the decreased cell viability and apoptosis induced by OGD/R, which is related to the attenuation of oxidative stress and the activation of mitophagy induced by OGD/R. More importantly, mitophagy inhibition blocked the mitigation of resveratrol in OGD/R-treated cells [110]. In the rat primary cortical neuron OGD/R model, Zuo et al. found phosphoglycerate mutase family member 5 (PGAM5) stimulated mitophagy via Drp-1, and miR-330 indirectly regulates mitochondrial autophagy by regulating PGAM5/DRP-1. Moreover, miR-330 downregulation improved the brain injury in the permanent middle cerebral artery occlusion (pMCAO) model [111].

#### 4.1.2. In Vivo

In 2013, Zhang et al. found that 3-methyladenine and Atg7 silencing reversed mitochondrial autophagy after reperfusion, suggesting that mitophagy underlies neuroprotection. Moreover, in the reperfusion phase, neuronal damage caused by ischemia can be aggravated *in vivo* and *in vitro* after treatment of Mdivi-1. Further investigation indicated PARK2 is transferred to mitochondria during reperfusion, and inhibition of PARK2 expression exacerbates ischemia-induced neuronal cell death ([91]). In a rat focal cerebral I/R injury model, the role of Parkin/DJ-1 mediated mitochondrial autophagy in adaptive neuroprotection after distal ischemia has been studied. The study suggests that remote ischemic posttreatment promoted mitophagy via the Parkin/DJ-1 pathway to mitigate focal cerebral I/R injury in rats [93]. In the mouse MCAO model, Yang et al. found cerebral I/R activated the clearance of impaired mitochondria in a BNIP3L/NIX-dependent manner, and BNIP3L knockout (bnip3l-/-) inhibited mitophagy, aggravating cerebral I/R injury in mice. When BNIP3L and Park2 genes were simultaneously knocked out, mice showed a synergistic mitochondrial autophagy deficiency during I/R therapy, but overexpression of BNIP3L produced a rescue effect on Park2^−/−^ mice [112]. Carfilzomib is a drug used for the treatment of multiple myeloma and can inhibit proteasomes. The study of Wu et al. indicated that carfilzomib could reverse the BNIP3L degradation and restore mitochondrial autophagy in the ischemic brain, which protected against brain injury after ischemia. Moreover, the protective effects were abolished in bnip3l-/- mice ([113]). In another *in vivo* study, MCAO-treated mice and oxygen-glucose deprivation- (OGD-) treated neurons, acidic postconditioning (APC) reinforced I/R-induced mitophagy, but the inhibition of mitophagy by a specific chemical inhibitor compromised the neuroprotective effect conferred by APC [114]. Interestingly, Shen et al. found APC activated mitochondrial autophagy through both cerebral ischemia *in vivo* and *in vitro*, but mitophagy and neuroprotection were abolished in PARK2 knockout mice, and APC-induced neuroprotection is related to PARK2-dependent mitophagy in the mouse model of MCAO [115]. Baicalin is a drug that can improve depression by inhibiting apoptosis ([116]). In the study of Li et al., the increased infarct size in hyperglycemia rats was prevented by Baicalin, and they proposed that activation of mitochondrial autophagy served as a therapeutic guideline for brain I/R in hyperglycemia ([94]). Activating transcription factor 4 (ATF4) can upregulate Parkin expression, which is a transcription factor involved in endoplasmic reticulum stress (ERS) ([117]). In a model of rat brain hypoxic-ischemic (H/I) injury, ATF4 overexpression induced by adeno-associated virus (AAV) was found to reduce infarct area and improve the neurological score. Mdivi-1, a mitochondrial autophagy inhibitor, significantly inhibited ATF4-mediated NLRP3 activation inhibition, and Parkin knockdown effectively reversed ATF4-mediated increased mitochondrial autophagy activity and NLRP3 activation inhibition [118]. There is evidence showing that pretreatment with electroacupuncture (EA) may induce rapid tolerance to cerebral ischemia ([119]). Moreover, studies found that EA can improve mitochondrial dysfunction induced by nitro/oxidative stress and inhibit mitochondrial autophagy through the PINK1/Parkin pathway, exerting neuroprotective effect against MCAO-induced brain injury [96]). Similarly, a tissue-type plasminogen activator (tPA) was found to exert a neuroprotective effect by inducing mitophagy in brain I/R injury, which was related to the activation of AMPK phosphorylation and subsequent enhanced FUNDC1 expression ([97]).

Some studies have confirmed that men are more likely than women to develop long-term cognitive deficits after neonatal hypoxic-ischemic encephalopathy (HIE) [120]. In a rat cerebral H/I model, there was a marked sex-specific difference with greater induction of mitophagy in females vs. males [121]. In acute cerebral ischemic (ACI) injury, Di et al. found that the induction of mitophagy by methylene blue (MB) mediated the protection against ACI injury, and thus, they proposed that MB may become a promising neuroprotective agent against acute ischemic stroke via activating mitophagy [122]. Similar to this study, in a traumatic brain injury (TBI) model, rapamycin (Rap, an mTOR inhibitor that can stimulate mitophagy) and NLRP3 inhibitor (MCC950) showed neuroprotection on TBI, while combined treatment with Rap and MCC950 exerted synergistic neuroprotective effects. These findings indicate that Rap-mediated activation of mitochondrial autophagy in combination with NLRP3 inflammasome may be useful for the treatment of TBI (Y. Chen et al., 2019).

Voltage-dependent anion channels (VDAC) are well known for the role of mitochondrial docking sites in promoting mitochondrial autophagy ([123]). In a rat subarachnoid hemorrhage (SAH) model induced by endovascular perforation, Li et al. studied the role of mitochondrial autophagy in cerebral injury 48 hours after SAH. They found that VDAC1 induced a significant increase of LC3-II after SAH, and the LC3-II was in turn significantly decreased after treatment with VDAC1 siRNA. Furthermore, they indicated that mitophagy played a significant role in the VDAC1-induced neuroprotection following SAH ([92]). In the same model, Cao et al. found melatonin alleviates the decline in brain function following SAH, which was ascribed to the activation of mitophagy and inhibition of ROS under SAH. Moreover, melatonin inhibits NLRP3 activation and weakens the secretion of proinflammatory cytokines following SAH. Their results suggest that melatonin alleviates brain damage after SAH through upregulated mitochondrial autophagy [98]. Interestingly, Sun et al. indicated that melatonin could increase the Nrf2 expression to activate mitophagy, which exerted neuroprotection against brain injury after SAH ([99]). This is consistent with the above results. Mitoquinone (MitoQ), a potent mitochondrial-targeting antioxidant, is effective in preventing mitochondrial dysfunction [124]. In a SAH animal model, it was found that MitoQ promoted mitochondrial autophagy after SAH through the Keap1/Nrf2/PHB2 pathway, alleviated mitochondrial oxidative stress-related neuronal apoptosis, and improved neurological function ([100]).

### 4.2. Inhibition of Mitophagy

Mitochondrial autophagy damage is considered to be a key link leading to many pathological conditions. However, there is evidence showing that overactivation of mitophagy is also harmful to cell hemostasis [74]. Therefore, treatment of stroke by inhibiting mitochondrial autophagy has also been studied.

#### 4.2.1. In Vitro

Carnosine (*β*-alanyl-l-histidine) is expressed in the central nervous system, which is an endogenous dipeptide. In a model of mouse permanent focal cerebral ischemia, carnosine robustly reduced brain damage after stroke [125, 126]. Baek et al. investigated whether carnosine protected against mitochondrial damage and mitophagy in ischemic stroke. They examined the levels of p-Drp1 and Parkin and confirmed the protective effect of carnosine against ischemia-induced neuronal mitophagy in primary cortical neurons. They found that the significantly increased expression of p-Drp1 and Parkin after ischemia was attenuated by carnosine [101]. It has been reported that oxygen-glucose deprivation/reperfusion (OGD/RP) may induce autophagy and mitophagy, and inhibition of mitochondrial calcium uniporter (MCU) can increase Ca^2+^ transport into the mitochondria, protecting the cerebral injury after I/R [127]. Interestingly, in the OGD/RP-treated SH-SY5Y cell model, Yu et al. found that MCU inhibition attenuated the OGD/RP-induced mitochondrial autophagy. The study has reported that MCU inhibition can downregulate OGD/RP-induced mitochondrial autophagy and protect neurons from I/R brain injury ([102]). Similar findings are also observed in mouse hippocampal neuronal cells (HT22 cells) treated by OGD/R *in vitro*. Deng et al. found that small nucleolar RNA host gene 14 (SNHG14) induced excessive mitochondrial autophagy through the Mir-182-5p/BINP3 axis and aggravated neuron damage. They proposed that appropriate mitochondrial autophagy had a protective effect on neurons [103].

#### 4.2.2. In Vivo

In the rat brain I/R injury model, increased production of peroxynitrite (ONOO-), mitochondrial recruitment of Drp1 (due to tyrosine nitration of Drp1 peptide), and PINK1/parkin-mediated mitochondrial autophagy activation were observed during reperfusion, and ONOO-induced Drp1 tyrosine nitration may help Drp1 mitochondria recruit and activate mitophagy. FeTMPyP is an ONOO- decomposition catalyst ([128]) and could significantly reverse the mitochondrial recruitment of Drp1, mitophagy activation, and cerebral damage. These findings were also confirmed in *in vitro* experiments [129]. In 2018, the same group found naringin (4′,5,7-trihydroxy-flavanone-7-rhamnoglucoside), a natural antioxidant, could reduce the formation of 3-nitrotyrosine in mitochondria and inhibit the transport of Parkin to mitochondria, suppressing mitophagic activation, which was related to the protective effect of naringin on the brain I/R injury [130]. Consistent with these results, Zhang et al. found that Radix Rehmanniae, a medicine, could decrease the ONOO--mediated mitophagic activation to improve neurological function and ameliorate cerebral infarction in the transient cerebral ischemia rat model ([131]). BNIP3 is considered to be a regulatory protein of mitochondrial autophagy. In animal models of neonatal I/H, BNIP3 and NIX are highly expressed in a “delayed” manner and are associated with delayed neuron loss after stroke. BNIP3 deficiency significantly reduced neuron mitochondrial autophagy but increased nonselective autophagy after I/H. After the silence of BNIP3, the expression of NIX was upregulated, but it failed to make up for the loss of BNIP3 in the process of activating excessive mitophagy [74]. Increasingly attention has been paid to glycine as an effective therapy for mitochondrial protection of the liver [132], and it may play a protective role in neurodegenerative diseases [133]. In 2019, Cai et al. investigated whether glycine protected against cerebral H/I injury. In the H/I model of rats, they found glycine exerted a protective effect against H/I injury of neonatal. They also reported that PINK1, BNIP3, and LC3 II/I levels were lower than normal in the HIE plus glycine group, and glycine administration downregulated the AMPK pathway in H/I injury and inhibited mitophagy, exerting neuroprotective effects ([134]).

## 5. Prospective

Recent studies have supported the idea that regulated mitochondrial autophagy has a protective effect on stroke, at least to a certain extent. Therefore, we explored the underlying mechanisms of mitophagy and the roles in the pathogenesis and treatment of stroke. Although many researches are devoted to revealing the connection between mitophagy and stroke, many questions remain unanswered.

Findings from available studies are still conflicting on whether the inhibition of mitophagy or the promotion of mitophagy is neuroprotective. As above, the balance between activation and inhibition of mitophagy is crucial for cellular physiology, and excessive inhibition or activation of mitophagy will be harmful to cellular physiology. Thus, restoring this balance should be the goal in the treatment of stroke. However, whether the mitophagy is activated or overactivated and how the mitophagy is activated should be elucidated in different stroke models because the status of mitophagy and the pathways related to mitophagic activation vary among studies.

As above mentioned, stroke can be defined as two categories: ischemic and hemorrhagic, of which ischemic stroke is the main type. Currently, most studies focus on ischemic stroke and mitophagy, and the association between hemorrhagic stroke and mitophagy is less studied. It has been reported that the mechanisms and pathological processes involved in ischemic stroke and hemorrhagic stroke are different. Therefore, findings from cerebral ischemia models may not be applicable for hemorrhage stroke. In addition, the role of mitophagy might vary in different stages of ischemic stroke, and the dynamic change of mitophagy in the brain after stroke should be further elucidated in more studies.

Various studies investigated the therapeutic effect of regulating mitophagy in different stroke models. Even though this issue has been extensively investigated in preclinical animal studies, no clinical evidence has been reported so far. This might be ascribed to the conflicting findings on the role of mitophagy in the pathogenesis of stroke or the complexity of pathways related to the regulation of mitophagy. In addition, the safety of a specific treatment should be evaluated before the assessment of therapies targeting mitophagy in clinical trials. Although some studies have investigated the therapies targeting mitophagy, the safety of a specific treatment, the pharmacokinetics of a specific drug, and the routes by which the drugs are administered are less investigated. Thus, currently, the investigations about the role of mitophagy in the pathogenesis of stroke are still in their infancy.

To sum up, increasing evidence has indicated that regulated mitophagy can exert neuroprotectives in stroke, although some issues about the role of mitophagy in stroke remain elucidated. Thus, manipulating mitophagy to maintain the integrity and homeostasis of mitochondria may help propose some strategies in the treatment of stroke.

## Figures and Tables

**Table 1:** Studies related to the role of mitophagy in the pathogenesis of stroke.

Authors	Animal model	Conclusion	Findings
Zhang et al. [91]	ptMCAO model/OGD of primary cortical neurons	Protective	Cerebral ischemia-reperfusion induces mitochondrial phagocytosis by causing Parkin to translocate from the cytoplasm to mitochondria.
Li et al. [92]	tMCAO mouse model	Protective	Rapamycin therapy alleviates mitochondrial dysfunction after cerebral ischemia, which is associated with mitophagy activation.
Zou et al. [93]	Global ischemia model/OGD of primary cortical neurons	Protective	Drp-1 protects against ischemic injury by facilitating the activity of the autophagic pathway and hence the rapid removal of damaged mitochondria.
Li et al. [94]	MCAO/R model/OGD/REP of PC12 cells	Protective	Baicalin could regulate mitochondrial function and protect against hyperglycemia-aggravated I/R injury
Chen et al. [95]	TBI model of mice/OGD of primary cortical neuronal	Protective	Rap-activated mitophagy may be beneficial for TBI treatment.
Wang et al. [96]	MCAO/R model	Protective	EA ameliorates nitro/oxidative stress-induced mitochondrial functional damage against neuronal injury in cerebral I/R.
Hu et al. (2020) [97]	OGD of primary cortical neuronal	Protective	Enhancing PINK1/Parkin-dependent mitophagy could improve mitochondrial turnover.
Cai et al. [98]	MCAO/R model/OGD of HT22 cells	Protective	TPA relies on fundC1-mediated mitophagy to repair mitochondrial function and reduce neuronal apoptosis.
Cao et al. [99]	SAH model	Protective	Melatonin played a protective role in post-SAH EBI according to upregulated mitophagy.
Sun et al. [100]	SAH model/SH-SY5Y and U251 cells	Protective	Mitophagy induced by melatonin provides protection against brain damage after SAH.
Zhang et al. [101]	SAH animal model	Protective	After SAH, mitoquinone activates mitophagy through the Keap1/Nrf2/PHB2 pathway to inhibit neuronal death related to oxidative stress.
Kumari et al. (2012) [102]	MCAO animal model	Harmful	Hyperglycemia enhances ischemia-induced mitochondrial imbalance.
Shi et al. [74]	Neonatal I/H model/OGD of primary cortical neurons	Harmful	Overactivation of BNIP3 leads to excessive mitochondrial autophagy with cell death.
Baek et al. [103]	MCAO model/(NMDA)-induced excitotoxicity of primary cortical neurons	Harmful	Carnosine inhibits ischemia-induced autophagy and mitochondrial damage, exerted neuroprotective.
Zhang et al. [91]	tMCAO model	Harmful	Inhibition of p38 can inhibit mitochondrial autophagy in cerebral ischemic injury.
Yu et al. [104]	OGD/RP model of SH-SY5Y cells	Harmful	Inhibition of MCU protects neurons from I/R damage.
Monda et al. (2019) [105]	MCAO animal model	Harmful	THC epigenetics improves cerebral vascular mitochondrial dysfunction in stroke.
Deng et al. [106]	OGD/RP of HT22 cells	Harmful	lncRNA SNHG14-induced excessive mitochondrial autophagy was associated with OGD/R-induced neuron damage.

Abbreviations: BHMT: betaine homocysteine methyltransferase; BNIP3L/NIX: BCL2/adenovirus E1B-interacting protein 3-like; ER: endoplasmic reticulum; HIF: hypoxia-inducible factor; H_2_: hydrogen; ICH: intracranial hemorrhage; I/R: ischemia-reperfusion; LC3: light chain 3; LIR: LC3-interacting region; 3-MA: 3-methyladenine; MCAO: middle cerebral artery occlusion; OGD/RP: oxygen-glucose deprivation/reperfusion; OGD/R: oxygen-glucose deprivation/reoxygenation; ONOO-: peroxynitrite; pMCAO: permanent middle cerebral artery occlusion; PINK1: tensin homolog- (PTEN-) induced kinase 1; SNHG14: small nucleolar RNA host gene 14; ST: stigmasterol.
